# Study of amino acids absorption and gut microbiome on consumption of pea protein blended with enzymes-probiotics supplement

**DOI:** 10.3389/fnut.2024.1307734

**Published:** 2024-01-23

**Authors:** Abhijit Rathi, Tejal Gaonkar, Debojyoti Dhar, Gopalakrishna Kallapura, Swati Jadhav

**Affiliations:** ^1^Human Nutrition Department, Advanced Enzymes Technologies Ltd., Louiswadi, Thane, India; ^2^Leucine Rich Bio Pvt. Ltd., Bangalore, KA, India

**Keywords:** amino acids, absorption, pea protein, digestion, gut microbiota, enzymes, probiotics

## Abstract

The current randomized, double-blind, crossover clinical trial was conducted to evaluate changes in the amino acid absorption and gut microbiota on consumption of pea protein supplemented with an enzymes-probiotics blend (Pepzyme Pro). A total of 15 healthy subjects were instructed to take test (pea protein + Pepzyme Pro) or placebo (pea protein + maltodextrin) for 15 days with a 30-day washout period. Blood samples were analyzed for plasma-free amino acids, insulin, and C-reactive protein (CRP). Additionally, nitrogen levels in urine and feces, along with the composition of gut microbiota, were evaluated. On day 15, the test arm showed a tendency to increase the rate of absorption and total absorption (AUC) of amino acids compared with the placebo arm, though the increase was statistically insignificant. In addition, 15-day test supplementation showed a tendency to reduce Tmax of all the amino acids (statistically insignificant except alanine, *p* = 0.021 and glycine, *p* = 0.023) in comparison with the placebo supplementation. There were no changes in urine and fecal nitrogen levels as well as serum CRP levels in the test and placebo arm. The increase in serum insulin level after 4 h was statistically significant in both arms, whereas the insulin level of the placebo and test arm at 4 h was not statistically different. Supplementation showed changes with respect to Archaea and few uncharacterized species but did not show statistically significant variations in microbiome profile at the higher taxonomic levels. A study with large sample size and detailed gut microbiome analysis is warranted to confirm the results statistically as well as to characterize altered species. However, the current study could provide an inkling of a positive alteration in protein digestibility, amino acid absorption, and gut microbiome with regular consumption of protein and enzymes-probiotics blend.

**Clinical Trial Registration:**clinicaltrials.gov/; identifier [CTRI/2021/10/037072].

## 1 Introduction

The increasing clinical evidence highlights the potential health benefits and significance of proteins. It has not only increased awareness but also heightened the popularity of protein supplements among athletes and recreationally active adults. Currently, numerous protein products are marketed as sports nutrients, muscle recovery supplements, medical and dietary formulations, weight management products, geriatric supplements, and maternal/infant dietary supplements (1–3). The sources of these dietary and therapeutic proteins could be from either animal or plant origins. Proteins from animal origin are considered complete proteins and are in high demand, but they come with some ethical and regulatory concerns. On the other hand, proteins from plant sources are abundantly available as good-quality proteins and overcome the ethical and regulatory issues that are associated with animal-sourced proteins (4, 5).

Pea protein is generally considered a high-quality protein as it satisfies FAO/WHO/UNU recommendations in terms of the availability of amino acids in a balanced ratio and the presence of all essential amino acids (EAA) except methionine (6). The digestible indispensable amino acid score (DIAAS) for the pea protein is nearly one, which makes it suitable to fulfill the amino acid requirements of the body (7). Furthermore, it has comparatively lower allergenic responses, negligible health controversies, and natural availability of biologically active peptides over the other plant proteins (7–9). Regular consumption of pea protein could offer a series of benefits including muscle growth, weight management, diabetes management, and healthy heart functions (10).

The nutritive benefits of the proteins mainly depend on the composition and bioavailability of the amino acids (11). Based on this fact, protein with high digestibility and bioavailability is always a preferred choice among athletes and recreationally active adults. Consumption of protein hydrolysate is another simplest and most widely used strategy to get the benefits of improved digestibility and bioavailability of the protein. On the other hand, these approaches come with the challenges such as limited sources of protein with high digestibility and possibilities of the presence of endogenous protease inhibitory peptides in the hydrolysate (12). Combining a protein of choice with a blend of enzymes and probiotics can be an alternate approach to enhance the protein digestibility and amino acid bioavailability. Earlier studies have shown that the co-ingestion of an exogenous enzyme blend along with plant protein increases protein digestibility (13) and reduces the difference between animal and plant protein in terms of nutritive benefits (14).

Previously, several clinical studies have demonstrated a positive impact of enzyme supplementation on protein digestion and absorption (15–17). Exogenous digestive enzymes can degrade dietary proteins simultaneously or sequentially with endogenous proteases to improve digestion and increase the availability of amino acids/smaller peptides for absorption (13). Furthermore, the effect of probiotics on the protein digestibility is of current interest. Jager et al. (18) have shown that the oral supplementation of probiotics along with the protein improved the postprandial changes in blood amino acids and can be a suitable approach to overcome compositional shortcomings of the plant protein. The positive influence of probiotics on the digestion of proteins is mainly associated with their ability to ameliorate the gut microbiota (19). This resulted in an improvement in protein digestion and its utilization, as well as a reduction in the harmful protein fermentation and associated health risks (20). Furthermore, Garcia et al. (21) hydrolyzed pea protein with proteases followed by fermentation with lactic acid bacteria and found an increase in the degree of hydrolysis and a reduction in the immunogenicity of pea protein.

Pepzyme Pro was used as a model blend of enzymes and probiotics in the current interventional study. The study was initiated with the hypothesis that the addition of blend of enzymes and probiotics in pea protein would increase the protein digestibility and bioavailability. Additionally, it would possibly contribute in positive alteration in the gut microbiota. Based on the stated hypothesis, this study aimed to evaluate the effect of supplementation of an enzymes-probiotics blend with pea protein on the digestion of proteins and the bioavailability of amino acids. Furthermore, the impact of supplementation on the gut microbiota was also under the scope of this study. Additionally, the safety assessment was done by measuring fecal and urine nitrogen content, as well as insulin and C-reactive protein (CRP) level.

## 2 Materials and methods

### 2.1 Study design and subjects selection

This prospective, interventional, double-blinded, randomized, placebo-controlled, and crossover study was conducted in conformity with ICH-GCP (E6 R2) guidelines, the Helsinki Declaration, and the local regulatory requirements (Indian GCP, Indian Council of Medical Research, and New Drugs and Clinical Trials Rules-2019). There were no further changes or amendments made after the approval of the protocol. The trial included 15 healthy adult subjects with an age range of 18–35 years, normal body weight (body mass index (BMI) of 19–24.99 kg/m^2^), and the willingness to provide written informed consent and comply with study instructions for its duration. Subjects with a known history of (i) smoking or tobacco consumption, (ii) clinically significant physiological, neurological, or psychiatric disease, (iii) organ transplantation or surgery in the past 6 months, (iv) known hypersensitivity or idiosyncratic reaction, intolerance to any ingredients in the formulation or any related product, as well as severe hypersensitivity reactions (like angioedema) to any drugs or food products, and/or (v) difficulty in donating blood were excluded from the study. Participants who met the necessary inclusion criteria were further encouraged not to change their current physical activity levels and to refrain from exercise for 24 h before starting the clinical trial.

### 2.2 Investigational product (IP)

The enzymes-probiotics blend (Pepzyme Pro) was obtained as a gift sample from Specialty Enzymes and Probiotics, Chino, USA, and was used as an IP. It is a commercial formulation of proteolytic enzymes (acid proteases from *Aspergillus niger*) and probiotics (*Bacillus coagulans* LBSC DSM (17654), *Bacillus clausii* 088AE (MCC 0538), *Bacillus subtilis* PLSSC (ATCCSD 7280), *Lactobacillus acidophilus* 033AE, and *Lactobacillus plantarum* 022AE (MCC 0537). A sachet of pea protein (30 g) with the enzymes-probiotics blend (1% of the protein) and maltodextrin (1% of the protein) was used as a test supplement and a placebo supplement, respectively. Maltodextrin was used as a placebo to nullify the effect of excipient (maltodextrin) present in the Pepzyme Pro. The labeling and packaging of the test and placebo were the same, except for the coded batch numbers mentioned on the sachet. The participants, investigators, and entire study team were blinded for the treatment allocation until the end of the study.

### 2.3 Study protocol and supplementation

The study schedule is provided in Table 1. The study comprised of initial screening, baseline testing, and two 15-day supplementation periods separated by a 30-day washout phase. Block randomization was done on online randomization tool^1^ using a pseudorandom number generator. All the subjects were instructed to open a sachet of supplement (test or placebo) daily in the morning and mix it in lukewarm water (300–500 mL). They were further instructed to drink supplement mixture daily in the morning on empty stomach. Occurrence of adverse events and concomitant medication were recorded throughout the trial period. Site visit was planned on days 1 and 15 of each supplementation period. Upon visit, each subject was instructed to consume their respective protein supplementation, and blood samples were withdrawn at 0, 0.5, 1, 2, 3, and 4 h for the analysis of plasma-free amino acids. On day 15 of both the supplementation period, the blood samples were withdrawn at 0 h and 4 h for measuring serum levels of insulin and CRP. Urine (24 h collection) and fecal samples were collected on days 1 and 15 on each supplementation period for the analysis of nitrogen content. In addition, collected fecal samples were also used for the gut microbiota analysis.

**Table 1:** Study design.

Assessment	Screening		Treatment period			Washout	Treatment period			Safety follow-up
		Baseline testing	Day 1	Day 2–14	Day 15	Day 16–30	Day 31	Day 32–44	Day 45	Day 46–60
Visit Site (*Overnight fasting)	X	X	X*		X*		X*		X*	
Written Informed Consent	X									
Inclusion/ Exclusion criteria	X		X							
Randomization			X							
Demographics	X									
Body height and weight	X	X	X		X		X		X	
Medical / surgical history	X									
Prior medication history	X									
Physical examination	X		X	X	X	X	X	X	X	X
Vital signs	X	X								X
Instructions for supplementation	X	X								
Instructions for dietary recording	X	X								
Dietary restriction	X	X								
Dietary record check			X		X		X		X	
Dietary recording				X		X		X		
Study drug administration (With/Without Supplement)			X	X	X		X	X	X	
Blood sampling assessment (0 h, 0.5 h, 1 h, 2 h, 3 h, 4 h)			X		X		X		X	
Blood analysis 0 and 4 h sampling (CRP and Insulin)					X				X	
Urine Analysis (24 h urine collection)			X		X		X		X	
Fecal Sample Analysis (fecal Nitrogen analysis)			X		X		X		X	
Gut microbiome analysis			X		X		X		X	
Adverse event recording	X	X	X	X	X	X	X	X	X	X
Concomitant medication review	X	X	X	X	X	X	X	X	X	X

### 2.4 Analysis

The concentrations of amino acids, insulin, and CRP in plasma were determined at Thyrocare Mumbai, India, using liquid chromatography tandem mass spectrometry method, electrochemiluminescence immunoassay, and immunoturbidimetry, respectively. Urine and fecal nitrogen content were analyzed by using Dumas combustion method. Leucine Rich Bio Pvt. Ltd., India, analyzed the gut microbiota of all the test samples using the shotgun microbiome sequencing method.

### 2.5 Gut microbiota analysis

#### 2.5.1 Sample collection

Stool samples were collected using Invitek Molecular Stool Collection Module [Cat. No. 1038111300, Berlin, Invitek Molecular GmbH]. All participants were instructed appropriately to use the kit for the sample collection. The samples once collected were shipped under room temperature to the processing unit for DNA extraction.

#### 2.5.2 DNA extraction

DNA from stool samples was extracted using QIAamp® Fast DNA Stool Mini (Cat No./ID: 51604, QIAGEN) following the manufacturer’s “Fast DNA Stool Mini Handbook” for fast purification of genomic DNA. Eluted DNA was collected in 1.5 mL DNA Lo-Bind microcentrifuge tubes, and the quantity and quality of DNA were assessed by Qubit 2.0 DNA HS Assay (Thermo Fisher, Massachusetts, USA) and NanoDrop® (Roche, USA) to meet the sequencing requirements.

#### 2.5.3 Metagenome sequencing

Whole metagenome sequencing of all the samples was performed using long-read sequencing technology. Briefly, the DNA library was prepared with the Ligation sequencing kit (SQK-LSK109) (Oxford Nanopore Technologies (ONT), Oxford, UK), then loaded onto a R9.4.1 MinION flow cell (FLO-MIN106), and sequenced on the ONT MinION Mk1B device (MIN-101B). Basecalling and demultiplexing of sequence reads were performed with Guppy v4.2.2 and with assistance from MinKNOW GUI v4.1.22. Raw sequencing reads were stored in FastQ format for further computational analysis. The upstream analysis involved measurement of quality checks and quality improvement, including but not limited to host [human] sequence removal. This was followed by the alignment of quality-processed reads to a reference database of microbial genomes. The raw and % normalized abundances of all the microorganisms identified within these samples, were quantified, and later used for downstream analysis involving various statistical measures. Data filtering and data normalization steps were performed for the removal of low-quality or uninformative features from raw abundance data to improve downstream statistical analysis. Taxonomic composition of communities across samples and comparing groups were analyzed for direct quantitative comparison of abundances. Both the alpha and beta diversity analyses were performed using the phyloseq package (22, 23), and the results were plotted as box and whisker plots for alpha diversity and PCoA plot for beta diversity.

Differential abundance (DA) analysis was performed with five different DA tools, *viz.*, univariate analysis (24), metagenomeSeq (25–27), EdgeR (v3.12) (28), DeSeq2 (29), and LEfSe (linear discriminant analysis effect size) (30). Once the DA analysis was performed using individual tools, we identified those microbial species that were called significantly differentially abundant in “consensus” by all five DA tools, ensuring the robustness of the DA characterization.

### 2.6 Efficacy and safety variables

Primary endpoints were set to assess the efficacy of enzymes-probiotics blend in the digestion and absorption of pea protein. It includes the estimation of bioavailability of plasma amino acids within 4 h of consumption of protein in placebo arm and test arm, and pairwise comparison between both the arms. Secondary endpoints were set to evaluate the changes in nitrogen level in urine and feces, changes in insulin and CRP levels in serum, and changes in fecal gut microbiome. Additionally, an assessment of adverse events was done to evaluate the safety of the enzymes-probiotics blend after oral supplementation.

### 2.7 Sample power and statistical analysis

Data were examined using SAS software, version 9.1, by keeping a 5% significance level (confidence interval 95%) and maintaining a minimum statistical power of 80%. Primary and secondary endpoints were analyzed separately. The concentration vs. time curve (AUC) for each amino acid was calculated at all available time points using the linear trapezoidal rule. The difference between test arm and placebo arm was statistically analyzed using Student’s t-test. All the data were represented as mean ± standard error (SE). *p* ≤ 0.05 denotes statistical significance unless specified.

## 3 Results

**Figure 1:**
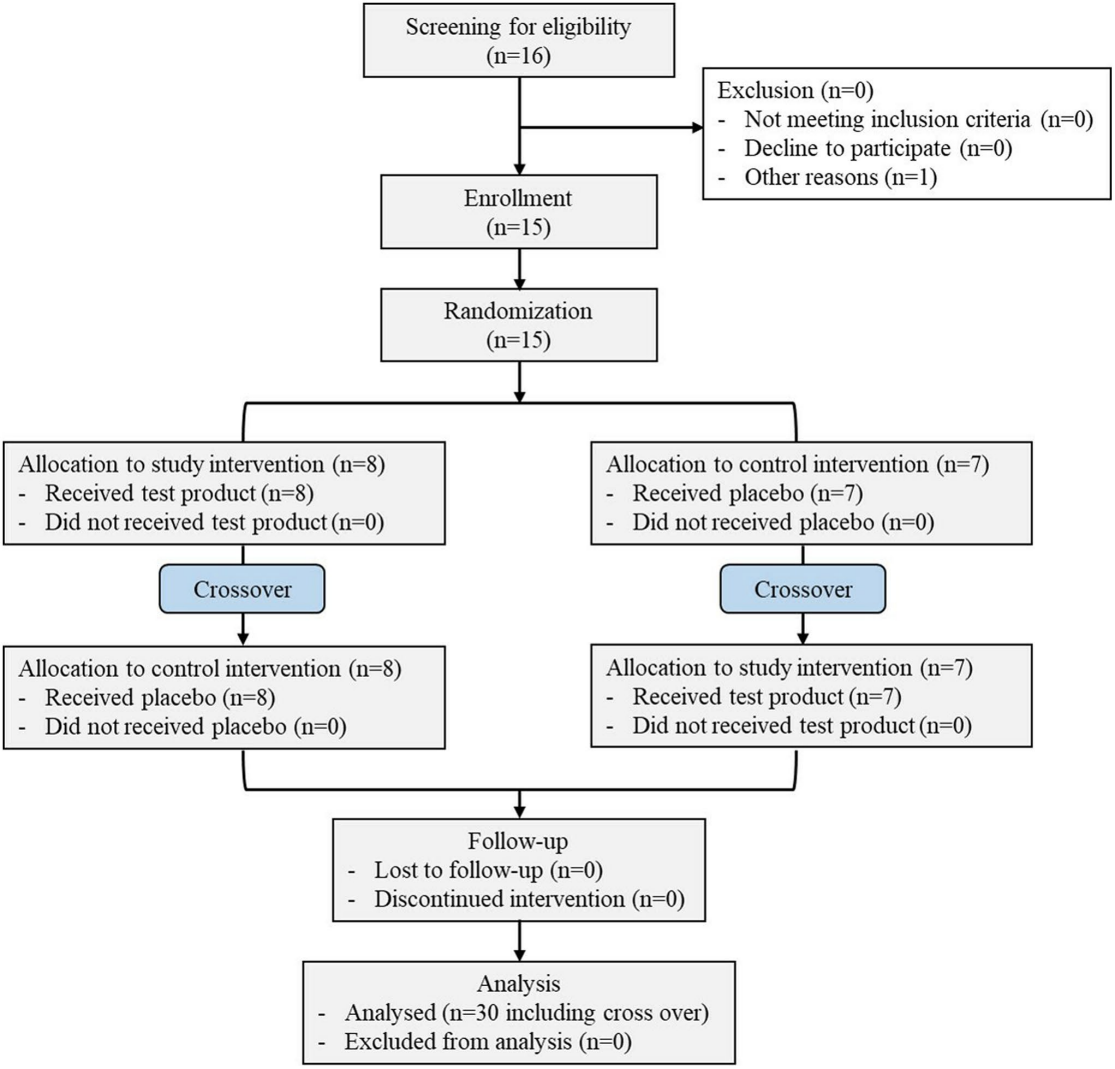
Schematic representation of the clinical study.

This randomized, double-blinded, crossover clinical study was initiated on 07 November 2021 and completed on 08 January 2022 (Figure 1) on 15 healthy subjects with average age of 21.40 ± 3.20 years. The other demographic details of the subjects, such as height, weight, and BMI, are presented in Table 2. All the patients completed the study.

**Table 2:** Subject characteristics at baseline.

**Gender**	**Male**
Age (Years)	21.4 ± 3.2
Height (cm)	171.6 ± 8
Weight (kg)	65.3 ± 9
BMI	22.1 ± 2.1

Values represented as mean ± standard deviation.

### 3.1 Primary endpoints

#### 3.1.1 Change in rate of amino acid absorption after consumption of pea protein

**Figure 2:**
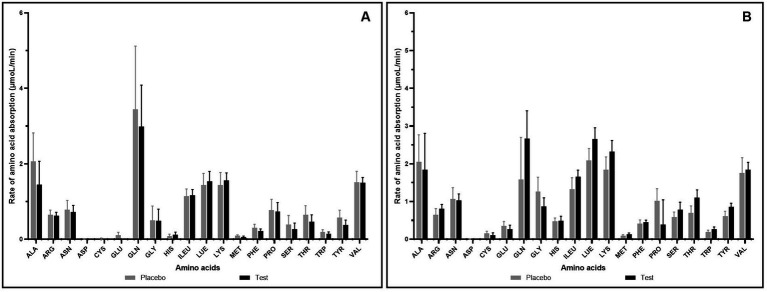
Rate of amino acid absorption (μmoL/min) after consumption of pea protein (placebo) *Vs* pea protein + enzymes-probiotics blend (test) on day 1 **(A)** and day 15 **(B)**. Values represented as mean ± standard error. Data were statistically analyzed using the Student’s *t-*test, and *p* ≤ 0.05 was considered statistically significant.

**Figure 3:**
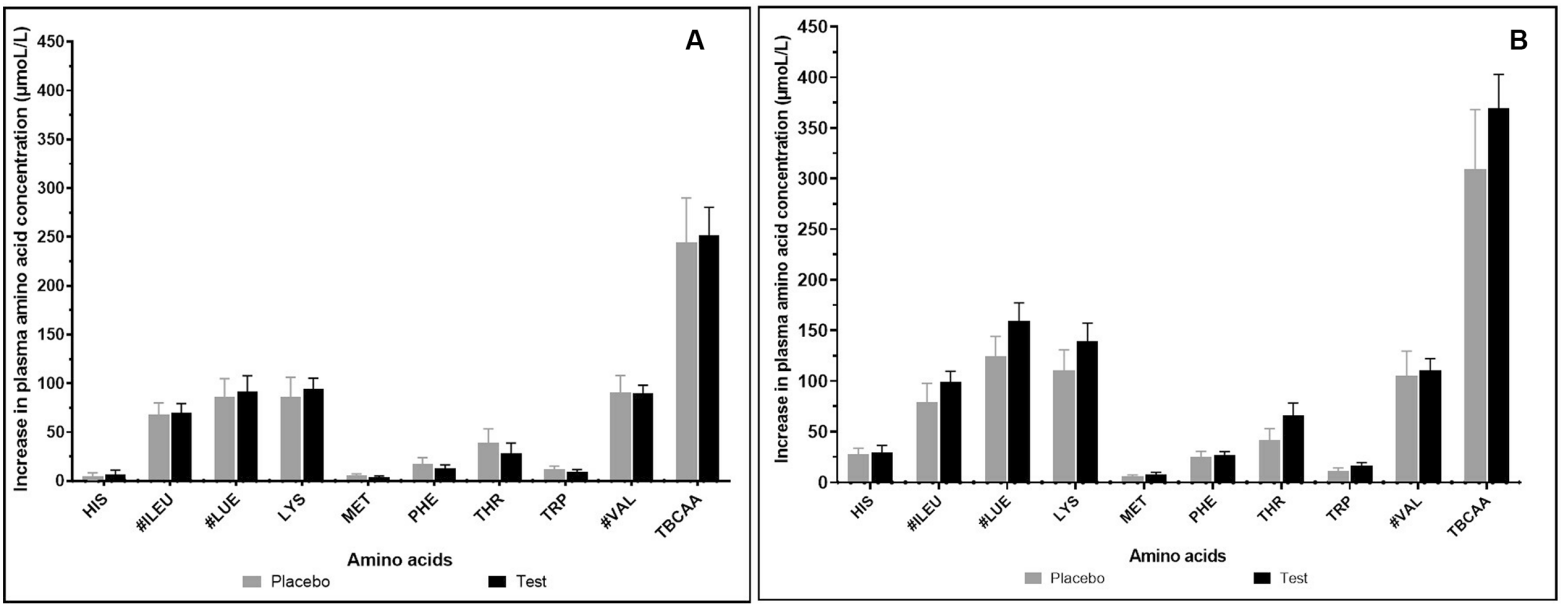
Increase in essential and branched chain amino acid concentration (μmol/L) after 1 h of consumption of pea protein (placebo) *Vs* pea protein + enzymes-probiotics blend (test) on day 1 **(A)** and day 15 **(B)**.Values represented as mean ± standard error. TBCAA: Total branched chain amino acids. # represents individual branched chain amino acid. Data were statistically analyzed using the Student’s *t-*test, and *p* ≤ 0.05 was considered statistically significant.

The effect of supplementation of enzymes-probiotics blend along with the pea protein for 15 days on the rate of amino acid absorption was measured for 4 h post-consumption on day 1 and day 15. On day 1, the rate of amino acid absorption in test and placebo arm was similar (Figure 2A). However, on day 15, variation was observed in the rate of amino acid absorption in the test and placebo arm (Figure 2B). After test supplementation, the rate of absorption of arginine, glutamine, isoleucine, leucine, lysine, methionine, serine, threonine, tryptophan, and tyrosine was positively altered by 1.25, 1.68, 1.25, 1.27, 1.26, 1.39, 1.35, 1.57, 1.44, and 1.41 folds, respectively (statistically insignificant). The analysis of essential amino acids (EAA) and branched chain amino acids (BCAA) illustrated no difference in the test and placebo arm on day 1. However, the appreciable difference though not statistically significant was observed in the increase of EAA and BCAA on day 15 within 1 h post-consumption (Figure 3). On day 15, test arm showed tendency to increase the plasma concentration of histidine (4.19%), isoleucine (25.09%), leucine (27.09%), lysine (26.37%), methionine (40.92%), phenylalanine (7.12%), threonine (57.21%), tryptophan (43.87%), and valine (5.36%), 1 h post-consumption of protein, over the placebo arm. In addition, overall absorption rate of EAA and BCAA on day 15 was in increasing trend than that of on day 1 in both placebo and test arm, though statistically insignificant.

#### 3.1.2 Absorption of total free amino acids after consumption of pea protein

The change in the absorption of free amino acids in test and placebo arm was analyzed by assessing the maximum plasma concentration (C_max_), the corresponding time (T_max_), and area under the curve (AUC). The AUC (concentration vs. time) was calculated for the plasma amino acids through the linear trapezoidal rule and using all available time points. C_max_ was defined as the highest observed concentration of amino acid, and T_max_ was the time when C_max_ was reached. On day 1, there was no noticeable difference in the AUC and C_max_ in the test and placebo arm, (Table 3A), whereas on day 15, test supplementation showed proclivity to increase the absorption of alanine, arginine, glutamine, glycine, isoleucine, leucine, lysine, methionine, phenylalanine, proline, serine, threonine, tryptophan, tyrosine, and valine as determined by the increase in the AUC and C_max_ values (Table 3B). Furthermore, the Tmax of all the amino acids in test and placebo arm was studied on day 1 and day 15. The T_max_ of tryptophan (4%) and tyrosine (11%) showed statistically insignificant decrease after consumption of test supplement on day 1 (Table 4A), whereas on day 15, test supplementation demonstrated the tendency to decrease T_max_ of the all amino acids except histidine (Table 4B). In particular, alanine (40%, *p* = 0.021) and glycine (40%, *p* = 0.023) showed statistically significant decrease in T_max_ after supplementation of the test product.

**Table 3A:** AUC and C_max_ for plasma amino acid concentration of placebo (Pea protein) and test (Pea protein + enzymes-probiotics blend) on day 1.

Amino acids	Pea protein + Placebo				Pea protein + Pepzyme pro				*p* value		Increase (%)	
	AUC (μmol*h/L)		C_max_ (μmol/L)		AUC (μmol*h/L)		C_max_ (μmol/L)		AUC	C_max_	AUC	C_max_
	Mean	SD	Mean	SD	Mean	SD	Mean	SD				
Alanine	2,730	1,084	790	285	2,481	906	748	294	0.500	0.692	−9.13	−5.35
Arginine	349	115	116	40	327	104	103	35	0.575	0.351	−6.52	−11.19
Asparagine	477	188	159	63	450	164	148	59	0.684	0.612	−5.57	−7.22
Aspartic acid	4.0	1.6	1.4	0.5	3.6	1.3	1.1	0.4	0.419	0.106	−10.84	−20.02
Cystine	168	59	54	20	119	32	37	10	0.009	0.007	−29.19	−30.89
Glutamine	172	50	63	17	166	58	56	18	0.759	0.286	−3.56	−11.07
Glutamic acid	5,341	3,986	1,488	1,081	5,290	3,253	1,501	901	0.970	0.970	−0.95	0.92
Glycine	1,533	610	459	176	1,362	403	410	133	0.373	0.391	−11.16	−10.83
Histidine	326	91	96	26	322	76	93	23	0.887	0.726	−1.35	−3.28
Isoleucine	458	139	154	51	453	145	149	50	0.918	0.772	−1.18	−3.51
Leucine	692	290	232	104	693	286	221	95	0.996	0.755	0.07	−4.94
Lysine	751	246	254	96	764	188	258	80	0.870	0.926	1.77	1.19
Methionine	99	54	32	16	97	40	31	12	0.882	0.758	−2.62	−5.00
Phenylalanine	281	100	85	32	249	74	75	23	0.321	0.326	−11.54	−11.91
Proline	1,416	451	403	132	1,423	574	401	166	0.971	0.971	0.49	−0.50
Serine	505	260	157	83	446	134	142	50	0.435	0.552	−11.82	−9.54
Threonine	590	264	181	87	566	180	177	66	0.771	0.884	−4.11	−2.29
Tryptophan	262	103	79	31	240	61	73	22	0.481	0.517	−8.43	−8.15
Tyrosine	343	167	112	57	322	71	105	26	0.651	0.683	−6.25	−5.97
Valine	1,020	322	312	96	1,050	290	307	78	0.791	0.857	2.94	−1.86
**Total**	17,518				16,821						−3.98	
**Total BCAA**	2,170				2,195						1.15	
**Total EAA**	4,480				4,432						−1.06	

**Table 3B:** AUC and C_max_ for plasma amino acid concentration of placebo (Pea protein) and test (Pea protein + enzymes-probiotics blend) on day 15.

**Amino acids**	**Pea protein + Placebo**				**Pea protein + Pepzyme pro**				***p* value**		**Increase (%)**	
	AUC (μmol*h/L)		**C**_**max**_ **(μmo/L)**		AUC (μmol*h/L)		**C**_**max**_ **(μmo/L)**		**AUC**	**C**_**max**_	**AUC**	**C**_**max**_
	**Mean**	**SD**	**Mean**	**SD**	**Mean**	**SD**	**Mean**	**SD**				
Alanine	2,457	1,472	712	407	2,860	1,392	852	438	0.447	0.371	16.43	19.68
Arginine	305	157	102	53	332	92	110	29	0.572	0.582	8.79	8.62
Asparagine	505	329	162	106	505	271	167	79	0.999	0.889	−0.04	2.95
Aspartic acid	4.0	2.4	1.2	0.7	3.8	2.0	1.2	0.6	0.805	0.858	−5.02	−3.64
Cystine	163	132	54	45	125	33	43	17	0.284	0.359	−23.60	−21.30
Glutamine	209	117	72	44	222	122	75	39	0.764	0.851	6.37	3.99
Glutamic acid	4,202	3,811	1,194	1,086	3,810	3,110	1,101	850	0.760	0.797	−9.33	−7.75
Glycine	1,621	701	469	198	1702	551	500	152	0.727	0.641	5.01	6.48
Histidine	359	120	117	47	340	120	110	47	0.661	0.688	−5.40	−5.95
Isoleucine	505	294	164	92	569	250	190	71	0.526	0.394	12.67	15.79
Leucine	824	384	275	142	913	345	321	122	0.508	0.348	10.84	16.79
Lysine	707	316	239	102	788	308	273	99	0.484	0.366	11.44	14.14
Methionine	77	29	25	9	85	31	29	12	0.467	0.280	10.47	16.93
Phenylalanine	244	118	75	35	280	109	86	30	0.396	0.358	14.69	14.93
Proline	1,157	913	333	253	1,331	895	407	305	0.602	0.474	15.05	22.30
Serine	517	167	159	55	589	238	186	82	0.343	0.291	14.01	17.29
Threonine	593	225	179	66	683	296	213	99	0.357	0.275	15.16	19.10
Tryptophan	227	81	65	23	249	84	75	22	0.463	0.286	9.87	13.91
Tyrosine	391	147	120	45	432	149	133	42	0.460	0.442	10.34	10.27
Valine	1,004	437	302	135	1,077	357	322	103	0.617	0.657	7.35	6.53
**Total**	16,072				16,897						5.14	
**Total BCAA**	2,333				2,560						9.73	
**Total EAA**	4,540				4,985						9.80	

**Table 4A:** T_max_ (h) for plasma amino acid concentration of placebo (pea protein) and test (Pea protein + enzymes-probiotics blend) on day 0.

**Amino acids**	**Pea protein + Placebo**		**Pea protein + Pepzyme pro**		***p* value**	**Decrease (%)**
	**Mean**	**SD**	**Mean**	**SD**		
Alanine	1.3	1.3	1.4	0.9	0.935	−3
Arginine	1.0	0.6	1.2	0.7	0.257	−28
Asparagine	1.2	1.3	1.2	0.8	>0.999	0
Aspartic acid	1.2	1.0	2.2	1.3	0.028	−86
Cystine	1.2	1.3	1.6	1.4	0.433	−32
Glutamine	1.0	0.7	1.8	1.5	0.061	−86
Glutamic acid	1.0	0.9	1.2	0.8	0.545	−20
Glycine	1.1	1.0	1.2	0.8	0.704	−12
Histidine	1.1	0.7	1.1	0.8	0.903	3
Isoleucine	1.2	0.7	1.5	0.6	0.153	−31
Leucine	1.4	1.1	1.7	0.5	0.464	−16
Lysine	1.2	0.9	1.4	0.7	0.670	−11
Methionine	0.9	0.6	1.1	0.7	0.483	−19
Phenylalanine	1.4	1.2	1.5	0.8	0.929	−2
Proline	1.9	1.4	1.7	1.1	0.719	9
Serine	0.9	0.6	1.4	0.8	0.067	−58
Threonine	1.3	1.1	1.6	1.0	0.342	−29
Tryptophan	1.6	1.3	1.5	0.8	0.865	4
Tyrosine	1.9	1.4	1.7	0.8	0.637	11
Valine	1.7	1.3	1.8	0.6	0.726	−8

**Table 4B:** T_max_ (h) for plasma amino acid concentration of placebo (Pea protein) and test (Pea protein + enzymes-probiotics blend) at day 15.

**Amino acids**	**Pea protein + Placebo**		**Pea protein + Pepzyme pro**		***p* value**	**Decrease (%)**
	**Mean**	**SD**	**Mean**	**SD**		
Alanine*	1.8	1	1.1	0.5	0.021	40
Arginine	1.7	1.1	1.4	0.6	0.372	18
Asparagine	1.5	0.9	1.2	0.6	0.256	22
Aspartic acid	1.9	1.2	1.6	1.2	0.413	19
Cystine	1.7	1.4	1.5	1.1	0.608	13
Glutamine	1.8	1.4	1.5	1.1	0.428	20
Glutamic acid	1.4	0.9	1.3	1	0.851	5
Glycine*	1.4	0.8	0.9	0.5	0.023	40
Histidine	1.3	0.9	1.6	1	0.391	−24
Isoleucine	1.7	0.9	1.3	0.6	0.247	20
Leucine	1.7	0.9	1.3	0.5	0.184	22
Lysine	1.5	0.9	1.2	0.4	0.27	20
Methionine	1.3	0.8	1.1	0.4	0.259	20
Phenylalanine	1.8	1	1.6	0.9	0.5	13
Proline	1.6	0.8	1.1	0.6	0.1	28
Serine	1.6	0.8	1.3	0.6	0.316	17
Threonine	1.5	0.9	1.2	0.6	0.232	22
Tryptophan	1.1	0.7	1.1	0.5	0.883	3
Tyrosine	2	0.8	1.5	0.7	0.12	23
Valine	1.9	0.9	1.7	0.7	0.658	7

* represents significant difference between placebo and test arm at *p* ≤ 0.05.

### 3.2 Secondary endpoints

#### 3.2.1 Safety assessment of enzymes-probiotics blend

During this clinical trial, no adverse events were recorded in both the study arms, ensuring safety upon consumption of supplement in human subjects.

#### 3.2.2 Change in nitrogen levels In fecal and urine samples

**Figure 4:**
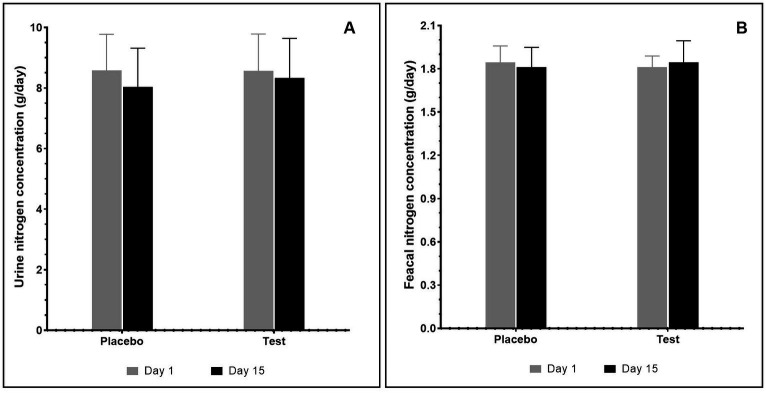
Total nitrogen content (g/day) after consumption of pea protein (placebo) and pea protein + enzymes-probiotics blend (test) present in urine **(A)** and fecal **(B)** samples. Values represented as mean ± standard error. Data were statistically analyzed using the Student’s *t-*test, and *p* ≤ 0.05 was considered statistically significant.

The effect of supplementation with pea proteins and enzymes-probiotics blend on fecal and urine nitrogen levels was evaluated in the test and placebo arms on days 1 and 15 of the study. The results indicated that supplementation did not alter the nitrogen levels in test compared with the placebo. Additionally, there was no statistically significant difference in the fecal nitrogen and urine nitrogen between the initial day (day 1) and the final day (day 15) of supplementation (Figure 4).

#### 3.2.3 Change in serum insulin and CRP level

**Figure 5:**
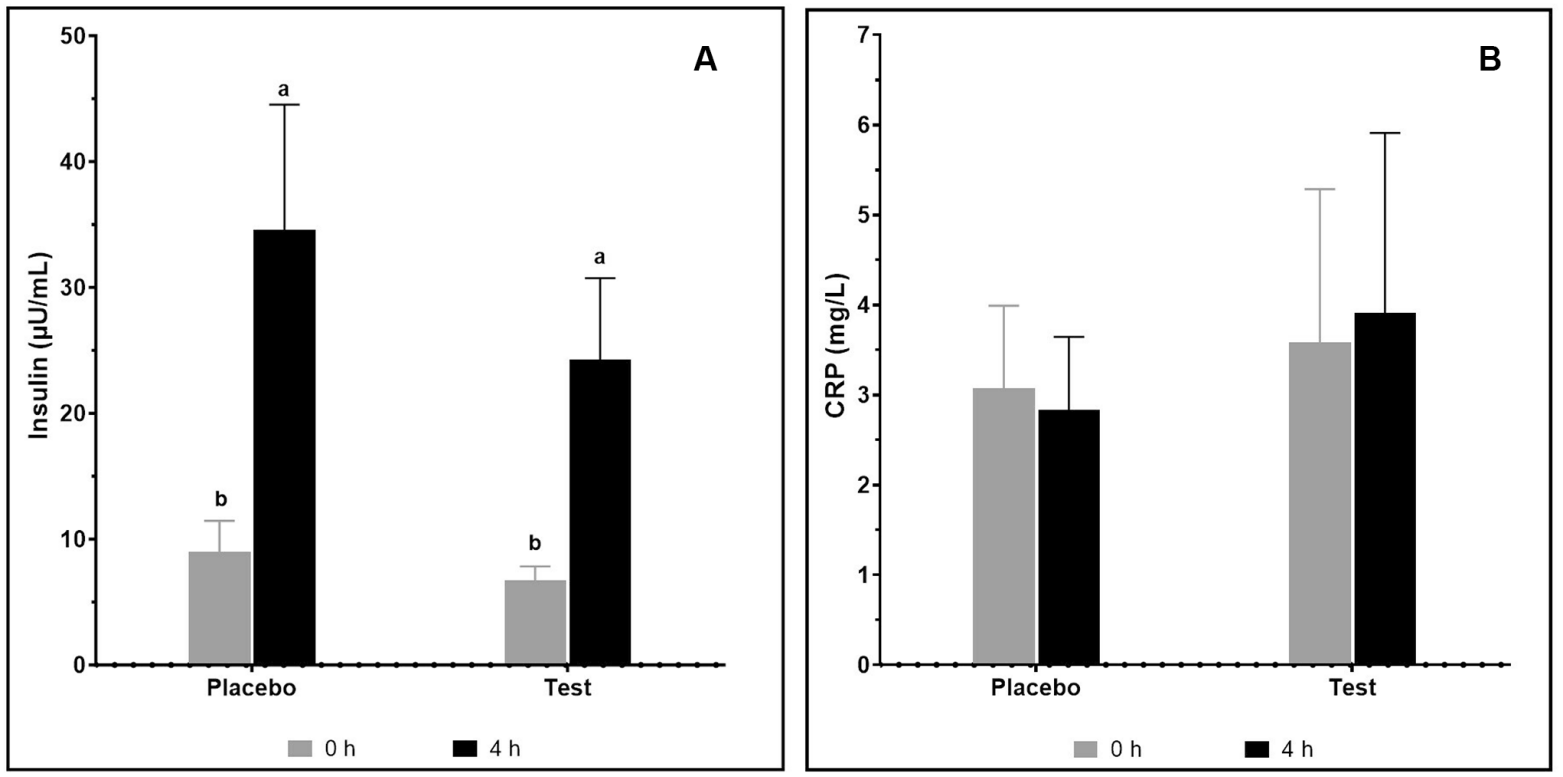
Insulin **(A)** and CRP **(B)** on consumption of pea protein (placebo) and pea protein + enzymes-probiotics blend (test) on day 15. Values represented as mean ± standard error. Data were statistically analyzed using the Student’s *t-*test. Lower letters placed on the bar represent statistical difference at *p* ≤ 0.05.

The changes in levels of serum insulin and CRP after the supplementation with pea proteins and enzymes-probiotics blend were quantified in the test and placebo arms on day 15 of the study (Figure 5). A considerable rise in the serum insulin level from 9.03 ± 2.43 μU/mL to 34.56 ± 9.98 μU/mL (*p* = 0.019) and 6.73 ± 1.11 μU/mL to 24.26 ± 6.48 μU/mL (*p* = 0.012) after 4 h of protein consumption was seen in the placebo and test arms, respectively. Consumption of protein has elevated the insulin level in both the placebo and test arms. The insulin level at 4 h in both arms was not statistically different. The CRP level did not show variation between the placebo and test arms as well as between the 0 h and 4 h samples, demonstrating the safety of the supplement.

#### 3.2.4 Changes in gut microbiome

The variations in the gut microbiome profile of the test arm after supplementation with pea protein and enzymes-probiotics blend on day 1 and day 15 were assessed by visualizing and characterizing for direct quantitative comparison of abundances. This was followed by alpha and beta diversity measurements, and then determining differentially abundant species across the comparing groups. As whole metagenome sequencing was performed, the profiling of all the microbial taxa (including fungi and viruses) within each sample was done successfully.

**Figure 6:**
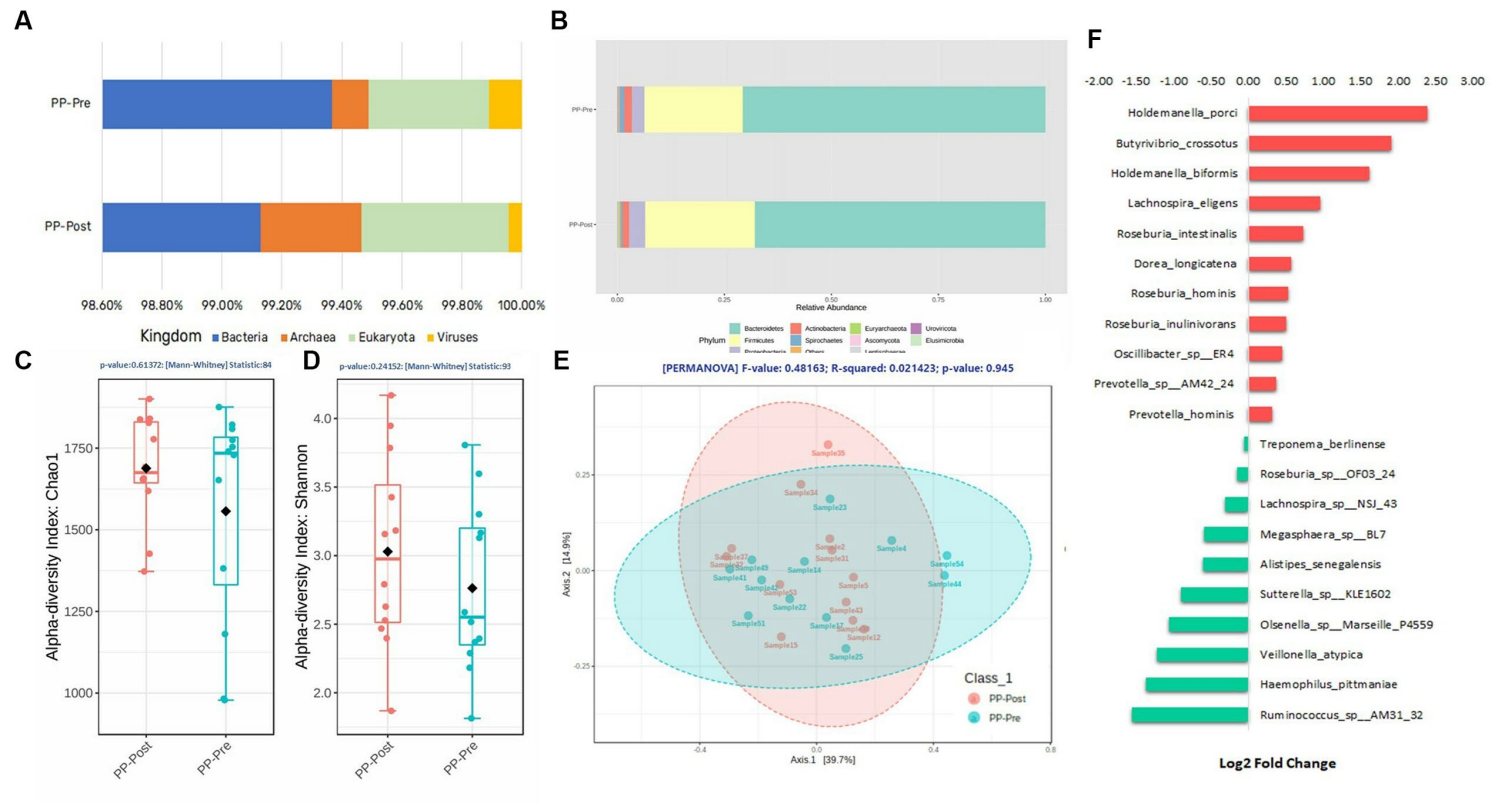
Composition of the gut microbiome after consumption of pea protein + enzymes-probiotics blend on day 1 (PP_Pre) and day 15 (PP_Post). **(A)** Quantitative comparison of abundances Kingdom level; **(B)** Quantitative comparison of abundances Phylum level; **(C)** Alpha Diversity—Chao1; **(D)** Alpha Diversity—Shannon Index; **(E)** Beta Diversity—Bray-Curtis distance, PERMANOVA; **(F)** Log2 Fold Change, of some of the most differentially abundant species.

**Figure 7:**
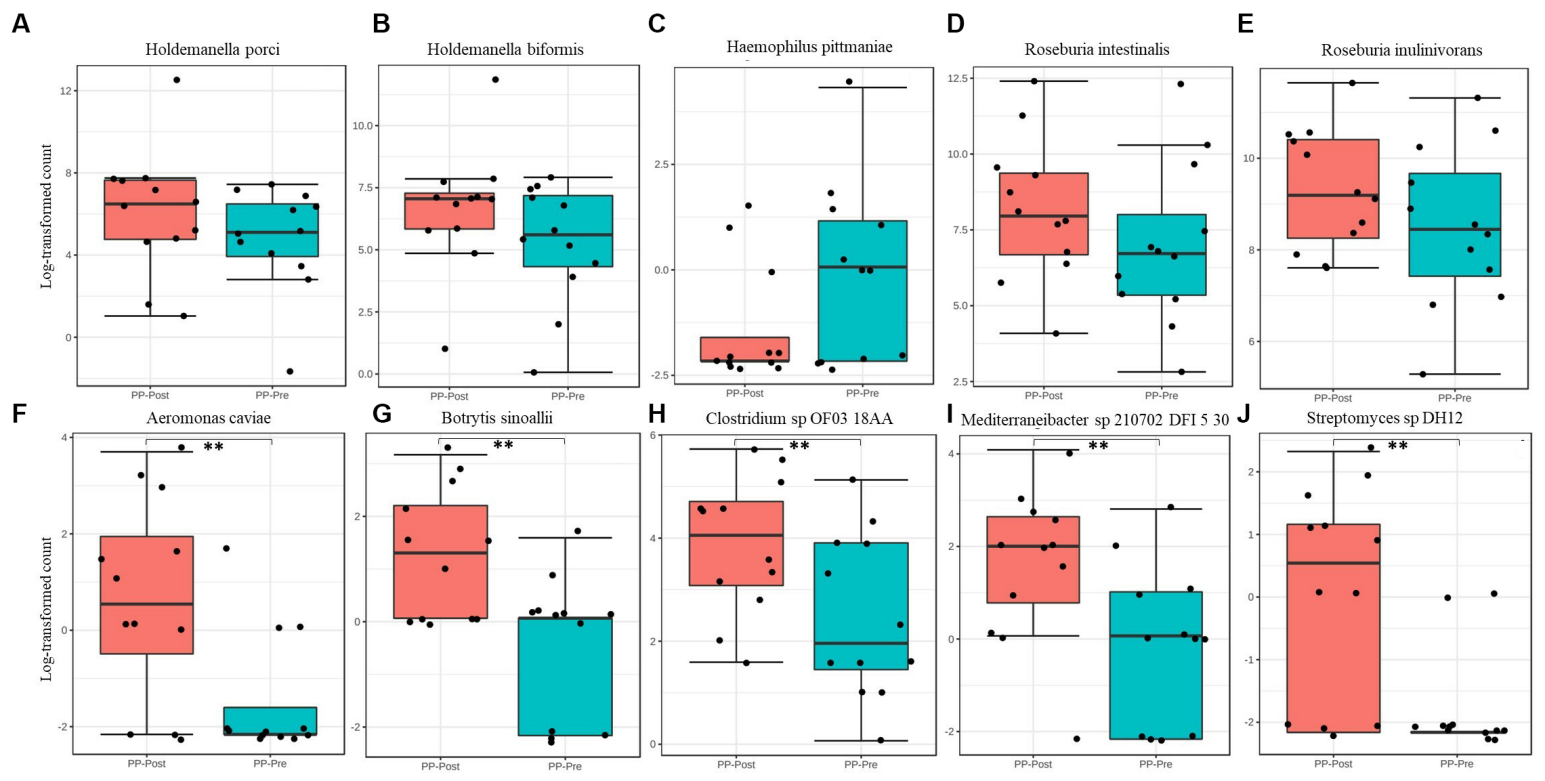
Differential abundance of species **(A)** *Holdemanella porci,* **(B)** *Holdemanella biformis,* **(C)** *Haemophilus pittmaniae,* **(D)** *Roseburia intestinalis,* **(E)** *Roseburia inulinivorans,* **(F)** *Aeromonas caviae,* **(G)** *Botrytis sinoallii,* **(H)** *Clostridium* sp. OF03 18AA, **(I)** *Mediterraneibacter* sp. 210,702 DFI 5 30, and **(J)** *Streptomyces* sp. DH12. ** represents significant difference between day 1 (PP_Pre) and day 15 (PP_Post) of supplementation at *p* ≤ 0.01.

The composition of microbial kingdom between day 1 (PP_Pre) and day 15 (PP_Post) of supplementation with pea protein and enzymes-probiotics blend is shown as stacked bar plots (Figure 6A). A slight but not statistically significant variation was observed in the microbial composition, particularly a small decline in the bacterial and viral populations post-consumption of test supplementation. The downtrend in bacterial and viral composition was from 99.37% (PP_Pre) to 99.13% (PP_Post) and 0.11% (PP_Pre) to 0.04% (PP_Post), respectively, after 15 days of supplementation with pea protein and enzymes-probiotics blend. Furthermore, the uptrend in archeal and eukaryotic abundance was from 0.12% (PP_Pre) to 0.33% (PP_Post), and 0.40% (PP_Pre) to 0.49% (PP_Post), respectively, after 15 days of supplementation. The relative abundance of this pattern can also be seen at phylum level (Figure 6B). The 15-day test supplementation showed the possibility of a downtrend in the abundance of Bacteroidetes and Proteobacteria, which may be due to a reduction in the abundance of Holdemanella (Holdemanella porci and Holdemanella biformis) (Figures 6F, 7A,B) and *Prevotella copri*. There was also a decline in *Haemophilus pittmaniae* of the phylum Proteobacteria (Figure 7C). The observed uptrend in the abundance of Firmicutes was largely due to an increase in the relative abundance of Roseburia (*Roseburia intestinalis*, *Roseburia hominis,* and *Roseburia inulinivorans*) (Figures 6F, 7D,E).

The measurement of alpha and beta diversity confirmed minor variations in diversities, but there were no statistically significant differences among the diversity measures on days 1 and 15 of the supplementation with pea protein and enzymes-probiotics blend. The Chao1 diversity displayed an increasing trend in the mean species richness in day 15 samples as compared to day 1 samples, with the diversity becoming more similar across all samples (lower deviation across samples) **(**Figure 6C). Furthermore, the Shannon diversity measure also showed increasing trend in diversities of day 15 samples with almost similar evenness as compared to day 1 samples (Figure 6D). The beta diversity measure by Bray-Curtis distance had not established any statistically significant difference between day 1 and day 15 of supplementation, even though it displayed clustering between the groups, represented by the ellipses (Figure 6E).

Though there were no statistically significant variations in microbiome profile at the higher taxonomic levels, we observed several specific changes at species level that were statistically, potentially, and functionally significant. Based on the consensus approach employed with five different DA tools, we could establish a total of five species to be significantly differentially abundant (*p < 0.05*) between day 1 and day 15. The comparative abundance plots of *Aeromonas caviae, Botrytis sinoallii, Clostridium* sp. *OF03 18AA, Mediterraneibacter* sp. *210,702 DFI 5 30, and Streptomyces* sp. *DH12* are displayed in Figures 7F-J.

## 4 Discussion

The present clinical trial was designed to evaluate the effect of the enzymes-probiotics blend on pea protein digestibility, amino acid bioavailability, and gut microbiota after regular consumption of high protein. Furthermore, the safety of the supplement was studied in terms of the adverse events, an alteration in serum insulin and CRP levels, as well as the changes in fecal and urine nitrogen levels.

Test supplementation showed an uptrend in the rate of amino acid absorption compared with the placebo on day 15, but not on day 1. The inability of the supplement to demonstrate positive effect on the amino acid absorption on day 1 illustrated that the external enzyme supplementation alone could not enhance the digestion and absorption of amino acids from the pea protein, but the probiotics aid the process. Consumption of the supplement for 15 consecutive days demonstrated the likelihood of augmenting the absorption of total amino acids (AUC) and maximum concentration (C_max_), as well as the tendency to reduce the time required to reach the maximum concentration (T_max_) in the blood. These results illustrated the possibility of enzymes-probiotics blend to positively alter the pea protein digestion and absorption. Previously, our *in vitro* study had showed that the co-ingestion of Pepzyme AG (containing acid proteases) with protein (whey, pea, and collagen) improved the protein digestion (13).

Probiotics are known to help digestion of protein by promoting healthy intestinal flora. They regulate and influence intestinal bacteria related to protein digestion. Some probiotics could also secrete proteolytic enzymes capable of hydrolyzing proteins and can also induce host digestive proteases and peptidases. They also reduce the production of toxic metabolites by controlling or inhibiting the fermentation of harmful proteins. Furthermore, probiotics also elevate the absorption of smaller peptides and amino acids by altering the abilities of the epithelium lining and promoting transport (20). The role of *Bacillus coagulans*, *Bacillus subtilis*, *Lactobacillus acidophilus,* and *Lactobacillus plantarum* in enhancing protein digestion has been well documented (18, 31–34). Previously, Jager et al. (18) documented the ability of probiotics to enhance the digestion of pea proteins. Garcia et al. (21) firstly hydrolyzed the pea proteins with digestive proteases and then fermented them using *Lactobacillus plantarum*, leading to production of smaller peptides which could be easily absorbed in gastrointestinal track. Furthermore, the enzymes-probiotics blend has been shown to boost the nutrient digestibility and to stabilize the microbial environment, thus promoting healthy gut in *Nile tilapia* (35). It is worth noticing that the digestion potential of probiotics could be augmented by blending them with exogenous proteases. In the current trial, 15-day supplementation of protein along with the enzymes-probiotics blend demonstrated an uptrend in rate of amino acid absorption and total amino acid absorption compared with the only protein supplementation. This could be possibly owed to the role of enzymes in digestion of protein and role of probiotics in regulating and influencing gut microbiota related to proteolysis.

The microbiota profiling data displayed an increasing trend in the abundance of Archaea, and specifically that of *Methanobrevibacter smithii* belonging to the phylum Euryarchaeota, post-test supplementation. This was interesting, as a previous study with whey protein supplementation reported an increase in Archaea diversity (36). *M. smithii* is highly prevalent microbes of the gut (37). In an earlier study, the presence of *M. smithii* had promoted calories intake from the diet in an animal model (38). The potential increase in the methanogenic archaea observed after supplementation of test for 15 days needs further detailed study with respect to the role of the Archaea and its functional impact. The supplementation also showed tendency to reduce the viral abundance and increase the abundance of Eukaryota (mainly fungi). At the phylum level, there was a downtrend in the abundance of Bacteroidetes and Proteobacteria and an uptrend in the Firmicutes (*Roseburia intestinalis* and *Roseburia inulinivorans*) on day 15. This can be functionally important as *Roseburia intestinalis* being one of the keystone species that influences the structure of the microbial communities (39), while *Roseburia inulinivorans* being one of the primary species associated with recovery of post-antibiotic exposure (40). On the other hand, pathogens like *Haemophilus pittmaniae*, belonging to phylum Proteobacteria, showed a tendency to reduce their abundance post-supplementation. However, none of these species were significantly differentially abundant across the groups. The comparative abundance of five species, namely *Aeromonas caviae, Botrytis sinoallii, Clostridium* sp. *OF03 18AA, Mediterraneibacter* sp. *210,702 DFI 5 30, and Streptomyces* sp. *DH12,* was estimated to be significantly different in the test on day 15 by at least 2 Log2Folds across the groups. Most of these microbiome species have been identified and characterized within the gut microbiome of humans recently. For instance, *Clostridium* sp. *OF03 18AA* and *Mediterraneibacter* sp. *210,702 DFI 5 30,* both belonging to the phylum Firmicutes, are relatively new and comes under the group of uncharacterized isolates of *Clostridium* (41) and *Mediterraneibacter*, respectively (42). Hence, their increase in abundance or its functional association post-supplementation of pea protein and enzymes-probiotics blend needs further evaluation. Of the five species, *Streptomyces* sp. *DH12* was perhaps the one with most potential to have a positive impact on the gut microbiome, with broad-spectrum antibacterial activity toward Gram-positive and Gram-negative pathogens, based on its genomic characterization (43). This was estimated to have increased by ~3-fold after supplementation with pea protein and an enzymes-probiotics blend. Though the statistically significant differences were not observed among the test and placebo group at phylum level of gut microbial population, the study provided inkling of possible positive effect which supplement might offer on long-term consumption.

Enzymes-probiotics supplementation was found to be safe in this trial as no adverse effects were recorded. Furthermore, it did not negatively affect the metabolism as shown by unaltered nitrogen levels of urine and feces as well as CRP on the initial and final day of the trial in both the placebo and test arms. This confirms the safety of the supplementation. The level of serum insulin was increased after consumption of pea protein in placebo as well as in test arm on day 15. Thondre et al. (44) has already documented the role of pea protein on the secretion of insulin. Though statistically insignificant, low levels of serum insulin in the test over the placebo arm may be attributed to the fixed time-point (0 h and 4 h) analysis. A potential decline in the T_max_ after 15-day test supplementation might be owing to earlier insulin spike in the blood. The time kinetics for the serum insulin levels after protein supplementation would definitely help to shed more light on the observed results. Although an increase in protein digestibility was seen in test arm, no statistically significant difference was found between test and placebo arms. The variation in the digestive processes and amino acid metabolism of each individual and relatively small subject size making it difficult to justify the results statistically.

## 5 Conclusion

The placebo-controlled, double-blinded, randomized, and crossover study conducted on 15 healthy subjects demonstrated the possible effect of the enzymes-probiotics blend (Pepzyme Pro) on the digestion of pea protein and gut health. Supplementation of the enzymes-probiotics blend along with the pea protein for 15 consecutive days provided an inkling of a possible uptrend in the rate of amino acid absorption, total amino acid absorption (AUC), C_max_, and a downtrend in the T_max_ compared with the placebo arm. Supplementation did not show statistically significant variations in microbiome profile at the higher taxonomic levels; expect changes observed with respect to Archaea and a few uncharacterized species. A detailed study related to the gut microbiome might be useful to characterize those species. The small sample size and metabolic variations had affected the statistical significance of the data. A study on a large population would be useful to shed more light on the details.

## Data availability statement

The original contributions presented in the study are publicly available. This data can be found at: https://www.ncbi.nlm.nih.gov/; PRJNA1020557.

## Ethics statement

The studies involving humans were approved by Institutional ethics committee Charak Hospital. The studies were conducted in accordance with the local legislation and institutional requirements. The participants provided their written informed consent to participate in this study.

## Author contributions

AR: Conceptualization, Methodology, Visualization, Writing – review & editing. TG: Data curation, Formal analysis, Visualization, Writing – original draft. DD: Data curation, Formal analysis, Writing – review & editing. GK: Data curation, Formal analysis, Writing – original draft. SJ: Conceptualization, Data curation, Formal analysis, Methodology, Visualization, Writing – original draft.

## Footnotes

^1^www.randomization.com

## References

[1] Karlund A Gómez-Gallego C Turpeinen A Palo-Oja O El-Nezami H Kolehmainen M. Protein supplements and their relation with nutrition, microbiota composition and health: is more protein always better for sportspeople? Nutrients. (2019) 11:829. doi: 10.3390/nu11040829, PMID: 31013719 PMC6521232

[2] Frestedt JL Zenk JL Kuskowski MA Ward LS Bastian ED. A whey-protein supplement increases fat loss and spares lean muscle in obese subjects: a randomized human clinical study. Nutr Metabol. (2008) 5:1–7. doi: 10.1186/1743-7075-5-8, PMID: 18371214 PMC2289832

[3] Herring C Bazer F Johnson G Wu G. Impacts of maternal dietary protein intake on fetal survival, growth, and development. Exp Biol Med. (2018) 243:525–33. doi: 10.1177/1535370218758275, PMID: 29466875 PMC5882021

[4] Hertzler S Lieblein-Boff J Weiler M Allgeier C. Plant proteins: assessing their nutritional quality and effects on health and physical function. Nutrients. (2020) 12:3704. doi: 10.3390/nu12123704, PMID: 33266120 PMC7760812

[5] Langyan S Yadava P Khan FN Dar ZA Singh R Kumar A. Sustaining protein nutrition through plant-based foods. Front Nutr. (2022) 8:1237. doi: 10.3389/fnut.2021.772573, PMID: 35118103 PMC8804093

[6] Gorissen S Crombag J Senden J Waterval W Bierau J Verdijk L. Protein content and amino acid composition of commercially available plant-based protein isolates. Amino Acids. (2018) 50:1685–95. doi: 10.1007/s00726-018-2640-5, PMID: 30167963 PMC6245118

[7] Guillin F Gaudichon C Guérin-Deremaux L Lefranc-Millot C Airinei G Khodorova N. Real ileal amino acid digestibility of pea protein compared to casein in healthy humans: a randomized trial. Am J Clin Nutr. (2021) 115:353–63. doi: 10.1093/ajcn/nqab354, PMID: 34665230

[8] Lu ZX He JF Zhang YC Bing DJ. Composition, physicochemical properties of pea protein and its application in functional foods. Crit Rev Food Sci Nutr. (2020) 60:2593–605. doi: 10.1080/10408398.2019.1651248, PMID: 31429319

[9] Ge J Sun C Corke H Gul K Gan R Fang Y. The health benefits, functional properties, modifications, and applications of pea (*Pisum sativum* L.) protein: current status, challenges, and perspectives. Compr Rev Food Sci Food Saf. (2020) 19:1835–76. doi: 10.1111/1541-4337.12573, PMID: 33337084

[10] Lonnie M Laurie I Myers M Horgan G Russell W Johnstone A. Exploring health-promoting attributes of plant proteins as a functional ingredient for the food sector: a systematic review of human interventional studies. Nutrients. (2020) 12:2291. doi: 10.3390/nu12082291, PMID: 32751677 PMC7468935

[11] Kumar M Tomar M Punia S Dhakane-Lad J Dhumal S Changan S. Plant-based proteins and their multifaceted industrial applications. LWT. (2022) 154:112620. doi: 10.1016/j.lwt.2021.112620

[12] Awosika T Aluko RE. Enzymatic pea protein hydrolysates are active trypsin and chymotrypsin inhibitors. Foods. (2019) 8:200. doi: 10.3390/foods8060200, PMID: 31185637 PMC6616451

[13] Jadhav S Gaonkar T Rathi A. In vitro gastrointestinal digestion of proteins in the presence of enzyme supplements: details of antioxidant and antidiabetic properties. LWT. (2021) 147:111650. doi: 10.1016/j.lwt.2021.111650

[14] Minevich J Olson MA Mannion JP Boublik JH McPherson JO Lowery RP. Digestive enzymes reduce quality differences between plant and animal proteins: a double-blind crossover study. J Int Soc Sports Nutr. (2015) 12:P26. doi: 10.1186/1550-2783-12-S1-P26

[15] Oben J Kothari S Anderson M. An open label study to determine the effects of an oral proteolytic enzyme system on whey protein concentrate metabolism in healthy males. J Int Society Sports Nutr. (2008) 5:10. doi: 10.1186/1550-2783-5-10, PMID: 18652668 PMC2500001

[16] Trehan A Verma MK Maheshwari S Khanna T Kumari P Singh H. An open-label clinical study to determine the effect of enhanced absorption formula (MB EnzymePro®) on the bioavailability of whey protein in healthy male subjects. J Food Process Technol. (2020) 11:820. doi: 10.35248/2157-7110.20.11.820

[17] Townsend J Morimune J Jones M Beuning C Haase A Boot C. The effect of ProHydrolase® on the amino acid and intramuscular anabolic signalling response to resistance exercise in trained males. Sports. (2020) 8:13. doi: 10.3390/sports8020013, PMID: 31978998 PMC7077235

[18] Jager R Zaragoza J Purpura M Iametti S Marengo M Tinsley G. Probiotic administration increases amino acid absorption from plant protein: a placebo-controlled, randomized, double-blind, multicenter, crossover study. Probiotics Antimicrob Proteins. (2020) 12:1330–9. doi: 10.1007/s12602-020-09656-5, PMID: 32358640 PMC7641926

[19] Hemarajata P Versalovic J. Effects of probiotics on gut microbiota: mechanisms of intestinal immunomodulation and neuromodulation. Ther Adv Gastroenterol. (2012) 6:39–51. doi: 10.1177/1756283x12459294, PMID: 23320049 PMC3539293

[20] Wang J Ji H. Influence of probiotics on dietary protein digestion and utilization in the gastrointestinal tract. Curr Protein Pept Sci. (2019) 20:125–31. doi: 10.2174/1389203719666180517100339, PMID: 29769003

[21] Garcia Arteaga V Demand V Kern K Strube A Szardenings M Muranyi I. Enzymatic hydrolysis and fermentation of pea protein isolate and its effects on antigenic proteins, functional properties, and sensory profile. Foods. (2022) 11:118. doi: 10.3390/foods11010118, PMID: 35010244 PMC8750503

[22] McMurdie PJ Holmes S. Phyloseq: an R package for reproducible interactive analysis and graphics of microbiome census data. PLoS One. (2013) 8:e61217. doi: 10.1371/journal.pone.0061217, PMID: 23630581 PMC3632530

[23] McMurdie PJ Holmes S. Shiny-phyloseq: web application for interactive microbiome analysis with provenance tracking. Bioinformatics. (2015) 31:282–3. doi: 10.1093/bioinformatics/btu616, PMID: 25262154 PMC4287943

[24] Calle ML. Statistical analysis of metagenomics data. Genomics Inform. (2019) 17:e6. doi: 10.5808/GI.2019.17.1.e6, PMID: 30929407 PMC6459172

[25] Paulson JN Pop M Bravo HC. metagenomeSeq: statistical analysis for sparse high-throughput sequencing. Bioconductor Package. (2013) 1:191. doi: 10.18129/B9.bioc.metagenomeSeq

[26] Paulson JN Talukder H Corrada BH. Longitudinal differential abundance analysis of microbial marker-gene surveys using smoothing splines. BioRxiv. (2017):099457. doi: 10.1101/099457

[27] Paulson JN Stine OC Bravo HC Pop M. Differential abundance analysis for microbial marker-gene surveys. Nat Methods. (2013) 10:1200–2. doi: 10.1038/nmeth.2658, PMID: 24076764 PMC4010126

[28] Robinson MD McCarthy DJ Smyth GK. edgeR: a bioconductor package for differential expression analysis of digital gene expression data. Bioinformatics. (2010) 26:139–40. doi: 10.1093/bioinformatics/btp616, PMID: 19910308 PMC2796818

[29] Love MI Huber W Anders S. Moderated estimation of fold change and dispersion for RNA-seq data with DESeq2. Genome Biol. (2014) 15:550. doi: 10.1186/s13059-014-0550-8, PMID: 25516281 PMC4302049

[30] Segata N Izard J Waldron L Gevers D Miropolsky L Garrett WS. Metagenomic biomarker discovery and explanation. Genome Biol. (2011) 12:R60. doi: 10.1186/gb-2011-12-6-r60, PMID: 21702898 PMC3218848

[31] Kumar VV Sudha KM Bennur S Dhanasekar KR. A prospective, randomized, open-label, placebo-controlled comparative study of *Bacillus coagulans* GBI-30, 6086 with digestive enzymes in improving indigestion in geriatric population. J Family Med Prim Care. (2020) 9:1108–12. doi: 10.4103/jfmpc.jfmpc_922_19, PMID: 32318476 PMC7113997

[32] Lin J Zeng Q Zhang C Song K Lu K Wang L. Effects of *Bacillus subtilis* supplementation in soybean meal-based diets on growth performance, diet digestibility and gut health in bullfrog *Lithobates catesbeianus*. Aquac Rep. (2020) 16:100273. doi: 10.1016/j.aqrep.2020.100273

[33] Pescuma M Hébert E Mozzi F Valdez G. Hydrolysis of whey proteins by *Lactobacillus acidophilus*, *Streptococcus thermophilus* and *Lactobacillus delbrueckii ssp. bulgaricus* grown in a chemically defined medium. J Appl Microbiol. (2007) 103:1738–46. doi: 10.1111/j.1365-2672.2007.03404.x 17953584

[34] Pranoto Y Anggrahini S Efendi Z. Effect of natural and *Lactobacillus plantarum* fermentation on in-vitro protein and starch digestibilities of sorghum flour. Food Biosci. (2013) 2:46–52. doi: 10.1016/j.fbio.2013.04.001

[35] Maas RM Verdegem MC Debnath S Marchal L Schrama JW. Effect of enzymes (phytase and xylanase), probiotics (*B. amyloliquefaciens*) and their combination on growth performance and nutrient utilisation in *Nile tilapia*. Aquaculture. (2021) 533:736226. doi: 10.1016/j.aquaculture.2020.736226

[36] Cronin O Barton W Skuse P Penney NC Garcia-Perez I Murphy EF. A prospective metagenomic and metabolomic analysis of the impact of exercise and/or whey protein supplementation on the gut microbiome of sedentary adults. MSystems. (2018) 3:e00044–18. doi: 10.1128/mSystems.00044-18, PMID: 29719871 PMC5915698

[37] Gaci N Borrel G Tottey W O'Toole PW Brugère JF. Archaea and the human gut: new beginning of an old story. World J Gastroenterol. (2014) 20:16062–78. doi: 10.3748/wjg.v20.i43.16062, PMID: 25473158 PMC4239492

[38] Samuel BS Gordon JI. A humanized gnotobiotic mouse model of host-archaeal-bacterial mutualism. Proc Natl Acad Sci U S A. (2006) 103:10011–6. doi: 10.1073/pnas.060218710, PMID: 16782812 PMC1479766

[39] Fisher CK Mehta P. Identifying keystone species in the human gut microbiome from metagenomic timeseries using sparse linear regression. PLoS One. (2014) 9:e102451. doi: 10.1371/journal.pone.0102451, PMID: 25054627 PMC4108331

[40] Chng KR Ghosh TS Tan YH Nandi T Lee IR Ng AH. Metagenome-wide association analysis identifies microbial determinants of post-antibiotic ecological recovery in the gut. Nat Ecol Evol. (2020) 4:1256–67. doi: 10.1038/s41559-020-1236-0 32632261

[41] Onate FP Roume H Almeida M. Recovery of metagenome-assembled genomes from a human fecal sample with pacific biosciences high-fidelity sequencing. Microbiol Resour Announc. (2022) 11:e00250–22. doi: 10.1128/mra.00250-22, PMID: 35532226 PMC9202402

[42] Schneider D Zühlke D Poehlein A Riedel K Daniel R. Metagenome-assembled genome sequences from different wastewater treatment stages in Germany. Microbiol Resour Announc. (2021) 10:e00504–21. doi: 10.1128/MRA.00504-21, PMID: 34236226 PMC8265224

[43] Jiao J Paterson J Busche T Rückert C Kalinowski J Harwani D. Draft genome sequence of Streptomyces sp. strain DH-12, a soilborne isolate from the Thar Desert with broad-spectrum antibacterial activity. Genome Announc. (2018) 6:e00108–18. doi: 10.1128/genomea.00108-18, PMID: 29496834 PMC5834332

[44] Thondre P Achebe I Sampson A Maher T Guérin-Deremaux L Lefranc-Millot C. Co-ingestion of NUTRALYS® pea protein and a high-carbohydrate beverage influences the glycaemic, insulinaemic, glucose-dependent insulinotropic polypeptide (GIP) and glucagon-like peptide-1 (GLP-1) responses: preliminary results of a randomised controlled trial. Eur J Nutr. (2021) 60:3085–93. doi: 10.1007/s00394-021-02481-8 33515092

